# What's the Use? *Disparate Purposes of U.S. Federal Bioethics Commissions*

**DOI:** 10.1002/hast.712

**Published:** 2017-05-22

**Authors:** Jenny Dyck Brian, Robert Cook‐Deegan

## Abstract

*In the forty‐year history of U.S. bioethics commissions, these government‐sanctioned forums have often demonstrated their power to address pressing problems and to enable policy change. For example, the National Commission for the Protection of Human Subjects of Biomedical and Behavioral Research, established in 1974, left a legacy of reports that were translated into regulations and had an enormous practical impact. And the 1982 report* Splicing Life, *by the President's Commission for the Study of Ethical Problems in Medicine and Biomedical and Behavioral Research, became the basis for the National Institutes of Health's Recombinant DNA Advisory Committee as well as for the Food and Drug Administration's developing “Points to Consider” when contemplating the introduction of recombinant DNA into human beings. Some efforts of bioethics commissions, however, are not tightly connected to policy change or to outcomes directly linked to a specific report. While direct policy impact is indeed a useful metric for government bioethics commissions, it is not their only legitimate utility. For instance, bioethics commissions can also be incubators for deliberation on a hot topic, giving policy‐makers time to think through options while the political heat has some time to dissipate. Or a bioethics commission may stake out a position that enables a politician to take action while not necessarily following its recommendations*.

## Comparative Analyses

In the late 1960s, Senator Walter Mondale held hearings about the wisdom of having a formal government mechanism to deal with the new technologies emerging from the life sciences. Talk of what became known as in vitro fertilization was in the air, rumors of human cloning circulated in the public media, and brain science was changing rapidly. Henry Beecher's 1966 *New England Journal of Medicine* article, “Ethics and Clinical Research,”^1^ raised concerns about research ethics but did not prompt immediate action. This was before stories about the Tuskegee syphilis study became public and attention turned to protection of people who were being studied in medical research. A few years later, recombinant DNA was debated in Congress and in the executive branch.

Senator Mondale's efforts in the Senate never elicited a House response, and the first bioethics commission was created in the reaction to Tuskegee. Tuskegee pushed the then‐nascent field of bioethics into the national spotlight, as a tool to address the pressing problems of research ethics. The formation of the commission encountered opposition, however. South African heart surgeon Christian Barnard and Stanford's Arthur Kornberg testified before Congress that pen‐wielding moralizers would use a government bioethics commission to obstruct progress in science and medicine. Others, though, including James Watson (codiscoverer of the structure of DNA), argued that many decisions about emerging biological technologies were not just technical decisions but required public deliberation. Senator Edward Kennedy took up the charge, and the House joined the effort this time. In 1974, President Gerald Ford signed the statute that established the National Commission for the Protection of Human Subjects of Biomedical and Behavioral Research.

Today, the National Commission is remembered mainly for its remarkable work on research ethics, a series of reports on vulnerable populations, culminating in the *Belmont Report* 2, which became the touchstone of federal regulations governing research that involved people as the objects of study. The National Commission left a legacy of reports that were translated into regulations and had an enormous practical impact, setting the template for the two‐pronged approach—independent review of research protocols by an institutional review board and compliance with criteria for informed consent and other research protections embedded in each protocol—that was embodied in the Code of Federal Regulations for federally sponsored research and for products and services regulated by the Food and Drug Administration. That basic framework continues into the current debate about revising the regulations created in the 1970s. The National Commission's deliberations thus led to reports that had direct impact on U.S. policy for how research is done. Its work scores high on both intellectual credibility and practical utility.

Despite the deep dive into research ethics, Senator Mondale's initial concern that biological technologies were racing ahead of the law and warranted systematic, national exploration and deliberation did not disappear. The National Commission did one report, largely outsourced to a contractor, that addressed emerging technologies, although few are aware it even exists.^3^ It is not a great report. It is barely coherent and runs to 568 pages. But one idea did shine through: that science was producing ideas and technologies that would not only change medicine but also spill over into our culture, challenging conceptions of what it means to be human. The report identified serious questions about how to manage powerful technologies affecting cells, organs, organisms, and society.

The idea that a national bioethics forum might address such questions did not die with the end of the National Commission. The Ethics Advisory Board, which existed from 1974 to 1979, addressed fetoscopy and in vitro fertilization. Prospects for genetically engineering human beings fell into the lap of the National Commission's successor, the President's Commission for the Study of Ethical Problems in Medicine and Biomedical and Behavioral Research. When religious leaders from the National Council of Churches, the Synagogue Council of America, and the U.S. Catholic Conference sent a letter to President Carter expressing concerns about a new era of fundamental danger triggered by the rapid growth of genetic engineering, he delegated the task of addressing their concerns to his President's Commission. Its report, *Splicing Life*,^4^ reviewed the arguments for and against genetic changes at a time when recombinant DNA was still relatively new and its use was contemplated for human gene therapy. That report became the subject of a House hearing before Representative Albert Gore, Jr., and the basis for the National Institutes of Health's Recombinant DNA Advisory Committee as well as for the Food and Drug Administration's developing “Points to Consider” when contemplating the introduction of recombinant DNA into human beings. This was another example of public bioethics leading to a practical outcome and formal policy response and, again, of earning high grades for both utility and scholarship.

These examples show the power of a government‐sanctioned bioethics forum to address pressing problems and to enable policy change. Another clear example is with the definition of death, with all states adopting statutes modeled on the President's Commission report *Defining Death*.^5^

Some efforts of bioethics commissions, however, are not tightly connected to policy change or to outcomes directly linked to a specific report. While direct policy impact is indeed a useful metric for government bioethics commissions, it is not their only legitimate utility.

Bioethics commissions can also be incubators for deliberation on a hot topic, giving policy‐makers time to think through options while the political heat has some time to dissipate. Or a bioethics commission may stake out a position that enables a politician to take action while not necessarily following its recommendations. President Clinton used the NIH's Human Embryo Research Panel's report in just this way, rejecting its most controversial recommendation to permit creation of human embryos for research under conditions where the embryos would never be implanted to become babies.^6^ While those on the panel may have felt that the president's reaction, coming just hours after the report was issued, undermined their work, the report had distinct political utility for the president: he used it as a foil, positioning himself to its right. He also adopted many of its other recommendations while deflecting political heat on the most controversial point. As noted by reports from the congressional Office of Technology Assessment and the Institute of Medicine, national bioethics commissions can serve as a forum to crystallize consensus or delineate points of disagreement; identify emerging issues; defuse controversy or delay decision‐making; propose regulations, develop guidelines, or formulate policy options; review implementation of existing laws and policies; aid judicial decision‐making; educate professionals and the public; and promote interdisciplinary research.^7^ There is no one role for such commissions.

Bioethical debate is one way to better understand the social, cultural, and political impacts of research and development. Federal, state, and local governments, pharmaceutical and biotechnology companies, hospitals, and professional associations have convened bioethics advisory bodies to help resolve dilemmas that require informed deliberation about ethical, legal, scientific, and economic considerations. But what, if any, authority—political or moral—do these committees have? Why should anyone listen to a bioethics commission?

Bioethics commissions have sometimes simply served as forums for gathering ideas, airing contending views that reflect moral pluralism, even conflict, but in a safe space. Deliberation, public education, and simply mediating a national debate have also been consistently valued. The President's Commission report *Securing Access to Health Care* 8 was among its most difficult, and it elicited dissent. But it also addressed an issue that had haunted American politics for most of a century—and still does, as witnessed by the partisan divides over the Affordable Care Act.

The last three presidential bioethics committees have all emphasized the value of public education. President Clinton's National Bioethics Advisory Committee interpreted its mandate to be one of both policy direction and public engagement, but like previous bioethics committees, it sought consensus. The President's Council on Bioethics, under President George W. Bush, functioned more like an advisory body, tasked with creating reports that surveyed the complex ethical dilemmas that arise with the use of various scientific technologies and medical treatments. Led by Leon Kass, the President's Council sought to foster and contribute to a wider debate, thus encouraging public deliberation. Through its work, it showed the value of clearly articulating competing arguments, and it raised valid concerns about foregrounding consensus for the sake of consensus. Indeed, President Obama's Presidential Commission for the Study of Bioethical Issues emphasized first and foremost a public engagement and education mission and sought to foster inclusive and open deliberations. There is tremendous value in mediating a national debate, even (or especially) if there is no receptivity within policy spaces.

These disparate uses of a bioethics commission are important and ought not to be dismissed in favor of a narrow emphasis on practical utility (in legislation or policy, for example). We face significant challenges in deciding how to structure and deliver bioethical analysis and advice. There have been nine federal bioethics committees in the United States, starting in 1974,^9^ suggesting that multiple administrations have believed that bioethics ought to and can contribute to key debates about science and society. But the ways in which the administrations have structured bioethics advice have differed significantly. We have now had two presidential bioethics committees in a row (one in a Republican administration and one in a Democratic administration) that have emphasized public deliberation over policy impact, opening space for us to imagine the broader range of what bioethics committees can and ought to accomplish. These disparate structures have been copied in other sectors and organizations. In the private sector, for example, although pharmaceutical and biotechnology companies have often sought the advice of individual bioethics consultants instead of creating committees of bioethics “experts,” a few companies, such as Advanced Cell Technology and SmithKline Beecham (now GlaxoSmithKline), have established standing bioethics committees with a variety of structures and functions.^10^ These committees have often directly replicated governmental bioethics committees: some have prioritized deliberation and achieved a better understanding of differing perspectives, and others have drafted ethics position papers and given concrete guidance to researchers in industry. The different strategies obviously achieved different—but not obviously better or worse—results for their respective organizations.

It's worth understanding and appreciating the range of possible structures for bioethics committees and the ways in which those structures enable and constrain different kinds of bioethical deliberation. The architects of bioethics committees, in nongovernmental as well as governmental settings, wrestle with critical concerns like whether committees ought to aim for consensus, how to avoid empty rhetoric and achieve political or policy impact, what the right kind of expertise is, what representation looks like, and how and when there is receptivity for a particular topic. A government‐sectioned committee with a mandate has an undeniable power. Linkage to government brings distinctive access to information, raises the entity's profile, and can have political utility even when a report does not lead directly to legislation, regulation, or other formal policy change. There are no set, agreed‐upon evaluative criteria for national bioethics committees, and a range of structures—a range that matches the diversity of bioethics work—has proven useful.
